# Be Kind to Children

**Published:** 1854-11

**Authors:** 


[For the Journal of Health.

### BE KIND TO CHILDREN.

Reader, be kind to children—then your name will be held by them, in after years, with a grateful remembrance—for impressions formed in childhood, though trifling in their nature, are almost always indelible.

If we speak an unkind word to a child, how soon a shade of gloom will steal over its little brow: and if they have really done something that deserves censure, remember they are but children, and you must expect they will do things inconsiderately; but it is your duty to forgive, and treat them with kindness, for we are satisfied, from what has come under our own observation, that kindness will control an obstinate child far better than severity. If that be true, be kind to children.

But perhaps a mother will say, "My child is so obstinate that I cannot help speaking to it harshly." But remember, mother, your harsh words have lost their power, and if you would control your child, let your voice be low and soft, as the Æolian harp: then the nerves of its little heart will vibrate pleasurably at the sound of its mother's voice; then even a look will control it more easily than all the unkind words imaginable.

We are ourselves often troubled with children, not our own, but we don't say to them, because they are a little in the way, go home, you are always where you ought not to be; but when we wish them to leave, we make them some trifling present, and invite them to retire, and come again at some future time. They most always cheerfully leave us, with their faces wreathed in smiles: then they are happy, and so are we.

Reader, be kind to children; then, when you are absent, they will always think of you with pleasure and delight.

MAHALA.

*Griswold's Mills.*

